# Inguinal and anorectal Lymphogranuloma Venereum: a case series from a sexually transmitted disease center in Rome, Italy

**DOI:** 10.1186/s12879-017-2484-8

**Published:** 2017-06-02

**Authors:** Alessandra Latini, Mauro Zaccarelli, Maria Grazia Paglia, Maria Gabriella Donà, Amalia Giglio, Domenico Moretto, Antonella Vulcano, Massimo Giuliani, Manuela Colafigli, Marina Ambrifi, Fulvia Pimpinelli, Antonio Cristaudo

**Affiliations:** 1 Clinic of Dermatology and Infectious Diseases (STI/HIV Unit), San Gallicano Dermatological Institute (IFO-IRCCS), Rome, Italy; 2 National Institute for the Infectious Diseases Lazzaro Spallanzani, Clinical Department, Rome, Italy; 3 National Institute for the Infectious Diseases Lazzaro Spallanzani,Microbiology Laboratory and Infectious Diseases Biorepository, Rome, Italy; 4 Clinical Pathology and Microbiology, San Gallicano Dermatological Institute (IFO-IRCCS), Rome, Italy

**Keywords:** Chlamydia, HIV, Lymphogranuloma Venereum, Men who have sex with men, Proctitis

## Abstract

**Background:**

Lymphogranuloma venereum (LGV) is a sexually transmitted infection caused by L1, L2, L3 serovars of *C. trachomatis* (CT). Since 2003, LGV cases have been increasing in Europe. Aim of this report is to describe the LGV cases diagnosed in the largest STI center in Rome, Italy, from 2000 to 2016. This report shows that two clinically and epidemiologically different series of cases exist, and that, at present, the ano-rectal LGV represents the clinical variant occurring more frequently among men having sex with men (MSM), particularly those HIV-infected.

**Case presentation:**

Ten cases of LGV were observed. Three were diagnosed in 2009 in HIV-negative heterosexuals patients that presented the classical genito-ulcerative form with lymphadenopathy. Seven cases were observed in 2015–2016 in HIV-infected MSM, that presented the rectal variant and L2b serovar infection; 4 of these had been misclassified as a chronic bowel disease.

Chlamydia infection was confirmed by CT-specific PCR (*ompA* gene nested PCR), followed by sequence analysis to identify the serovar.

All the patients were treated with doxycycline for 3 weeks, obtaining a complete response with healing of both clinical symptoms and dermatological lesions.

**Conclusions:**

Our findings suggest that, in case of persistent rectal symptoms in HIV-infected MSM, LGV should be taken into account and investigated through molecular analyses, in order to achieve a correct diagnosis and management of the patients.

## Background

Lymphogranuloma venereum (LGV) is a sexually transmitted disease (STD) caused by L1-L2-L3 serovars of *Chlamydia trachomatis* (CT). In the past century, LGV was diffused in tropical areas, including Africa, Asia and the Caribbean and only sporadic cases were described in Europe [1]. Unexpectedly, since 2003, some outbreaks of LGV have been reported in Western Europe, predominantly among HIV-infected men who have sex with men (MSM), as part of the increase in incidence of other STDs, such as syphilis, gonorrhoea, and type C hepatitis [2–4]. These outbreaks of LGV have been mainly described as ano-rectal forms determined by the serovar L of CT.

From 2004 to 2013, a total of 4761 cases of LGV were reported from 11 of the 32 European countries involved in the European STI Surveillance Network, of which 77 from Italy. In 2013, the reported LGV cases in the European Union were 1043, and they were mainly diagnosed in the age group 35–44 years (36.0%), in MSM (98.5%) and in HIV-1 infected individuals (62.0%) [5].

Laboratory investigations have a crucial role in the identification of LGV cases, but the availability of a reliable and simple method to discriminate between LGV and non-LGV CT infections is still challenging. Nucleic acid amplification techniques (NAATs) are sensitive and specific, but they are not CE marked for all types of sample required to make diagnosis of extra-genital LGV, e.g., rectal samples. Therefore, the use of in-house CT molecular typing tests instead of commercial assays is widespread [6].

Moreover, LGV may now occur with clinical pictures that can make the disease difficult to be recognized, particularly in non-specialistic medical areas, so that LGV diagnosis is often delayed or misclassified [7, 8].

The difficulty to have standardized and widely used molecular assays and a common clinical approach to identify LGV cases seems to suggest that the reported cases in Europe represent only the tip of the iceberg of a relevant and increasing epidemiological phenomenon.

In the last few years, in our STI center, which represents the largest one in Rome, Italy, we have increasingly observed the ano-rectal clinical variant of LGV among HIV-positive MSM. Aim of the present report is to review the experience of our STI center regarding LGV in the last 17 years and to describe the characteristics of the LGV cases recently diagnosed compared to those observed previously.

## Case presentation

All confirmed cases of LGV diagnosed after the year 2000 were retrieved from the electronic clinical archive of the STI center of the San Gallicano Dermatological Institute of Rome, Italy. Clinical and socio-demographic data were collected from the medical records.

From 2000 to 2016, 10 cases of LGV were diagnosed: 3 in 2009 and 7 in 2015–2016. Only one case was diagnosed in a woman. Out of the 9 cases observed in men, 7 were observed in HIV-infected MSM. All but two patients were Italian.

The three LGV cases observed in heterosexual individuals were diagnosed in 2009: a young couple from Morocco (a 22-year-old girl and a 26-year-old man) and a 60-year-old Italian man.

All the three patients were referred to our STI center by their general medical practitioner because of the onset of genital symptoms worsened during the previous month.

**Fig. 1:**
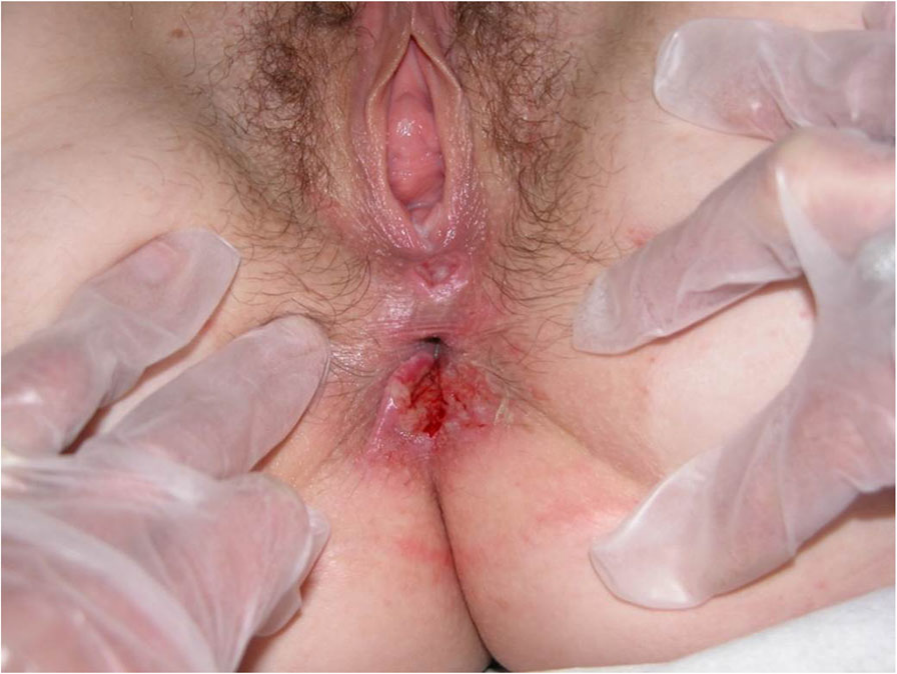
Ulcerative, painless lesion in a 22 year-old woman, wife of a man who was also diagnosed with LGV

**Fig. 2:**
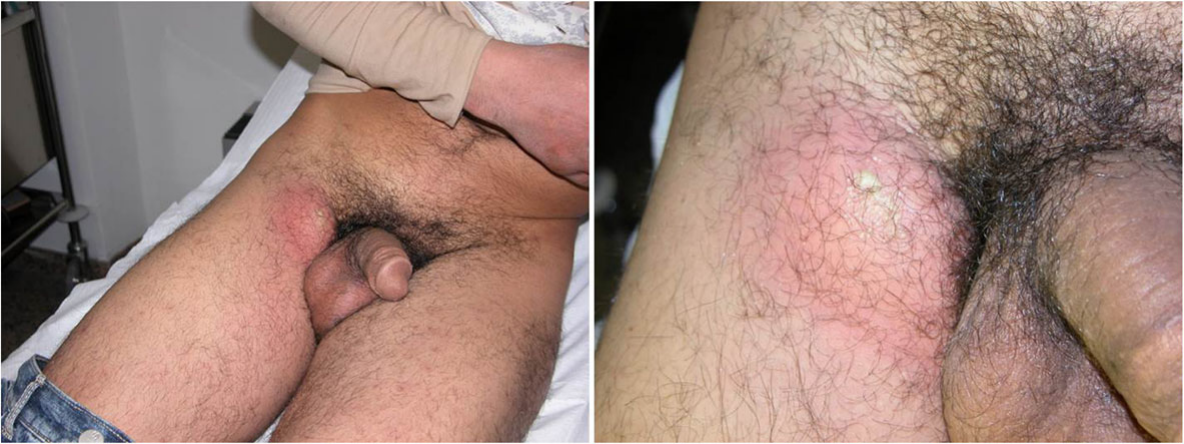
Bubo in a 26 year-old man with cutaneous microabscesses and “watering can sign” aspect

**Fig. 3:**
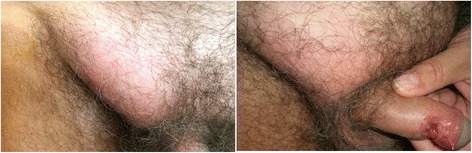
Ulcerative, painless penile lesion and inguinal lymphadenopathy (it is possible to see the “groove sign”) in a 60 year-old heterosexual man

The woman presented an ano-genital ulcerative lesion (Fig. 1), while her partner showed an inguinal lymphadenopathy. He presented additional constitutional symptoms, such as fever, fatigue and a severe unilateral purulent lymphadenitis (inguinal syndrome or “bubo”) (Fig. 2). The Italian heterosexual patient had an ulcerative, painless lesion at the rod side surface of the penis and a unilateral inguinal lymphadenopathy (Fig. 3).

**Fig. 4:**
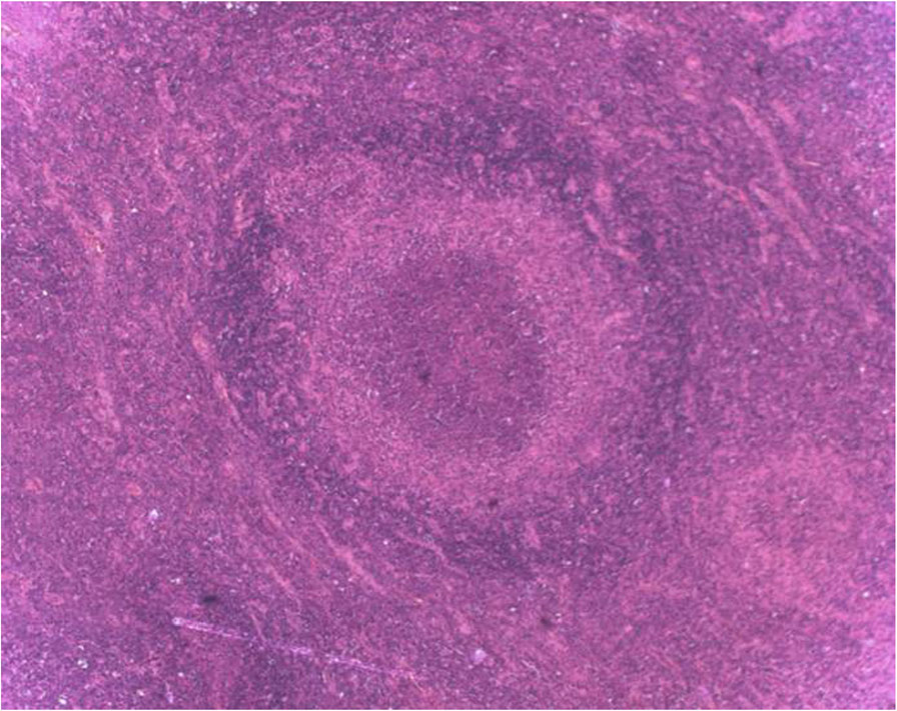
Hematoxylin and eosin stained histological section of a lymph node, with areas of necrosis and “starry abscesses”

These three patients were negative at serological tests for HIV and syphilis. LGV diagnosis was based on clinical and molecular evidence. In fact, CT-PCR (Anyplex™ CT/NG Real-time Detection 3.1 Assay, Seegene, Seoul, Korea), carried out on samples collected from the ulcerative genital lesions from the immigrant girl, penile ulcer from the Italian man and from the purulent discharge that flowed from the inguinal lymph node of the Moroccan patient, allowed to identify CT-DNA. In the 60-year-old man, LGV was also confirmed by the histological examination of the inguinal lymphnode. The presence of areas of necrosis or “starry abscesses” was consistent with LGV diagnosis (Fig. 4).

The 7 cases in HIV-infected MSM were diagnosed in 2015 (*n* = 3) and in 2016 (*n* = 4). These patients, of 25-40 years of age, were on steady combination antiretroviral therapy (cART) with an undetectable HIV-1 viral load, good immunological response and were in general good health. An acute proctitis was the initial clinical manifestation for all the MSM. The symptoms consisted of rectal tenesmus, mucous and blood secretion, and rectal pain during receptive anal intercourses. Three HIV-positive patients had been presenting the above mentioned symptoms in the previous month and were referred to our clinic by their general practitioners. The others had been presenting persistent ano-rectal and gastroenterological symptoms for 1–3 months. They were firstly referred to gastroenterologists and had undergone to colonoscopy with biopsies, all indicating inflammatory bowel diseases. All these patients had received local endo-rectal and systemic anti-inflammatory therapy without benefit, with a delay in the diagnosis of two to three months. CT-PCR (Anyplex™ CT/NG Real-time Detection 3.1 Assay) on rectal swabs was positive for CT-DNA and genotyping (performed by sequence analyses of a 990 bp fragment of the *ompA* gene [8]) revealed L2b serovar in all these patients.

Five MSM referred a history of specific anal practices with occasional partners during the previous year, such as fisting, sharing of sex toys and the use of chem-sex.

All the patients were treated with 100 mg/BID doxycycline for 3 weeks, following the recommendations of the European Guidelines [9]. A complete response with healing of both clinical symptoms and dermatological lesions was reached by all patients. They were all followed up 6 months after completing the treatment. No clinical signs and symptoms possibly related to LGV were reported.

## Discussion and conclusions

European Guidelines on Chlamydia control recommend that the diagnosis of LGV is based on clinical suspicion, epidemiologic information and differential diagnosis for procto-colitis, inguinal lymphadenopathy, or genital or rectal ulcers [10].

In our experience, the cases observed in the heterosexual individuals presented with the classic clinical manifestation, i.e., ano-genital ulcerative lesions and lymphadenopathy. Differently, the cases observed in the last 2 years, all occurring in HIV-infected MSM, presented with rectal symptoms. It is important to emphasize that during the period 2010–2014, no cases of LGV have been observed in our STI center, which is the largest in Rome. On the one hand, the complete absence of diagnoses in this time period might be due to the fact that many diagnoses have been missed because of a low awareness of the clinicians for this disease, which is rare in EU. On the other hand, this fact seems to suggest an epidemiological distance between the two clinically different series of cases, and a recent circulation of CT L2b serovar among MSM living in Rome.

Scarce data are available in Italy on the burden of LGV. In 2008 and 2014 two case reports have described anecdotal cases, both in MSM [11, 12]. Moreover, to our knowledge, between 2009 and 2014 only two studies have been conducted in Italy to assess the prevalence of anal CT Lb2 serovar infection among MSM. The prevalence of this serovar among asymptomatic individuals attending urban STI Clinic in Turin and Bologna was 1.4% and13.1%, respectively [7, 13]. One of these Italian studies also showed CT-DNA in 9.4% of 2660 anal swabs [7].

Generally, the individuals with anal CT Lb2 serovar infection show a history of neglected symptoms at anal level. In fact, signs and severe discomfort related to long lasting proctitis were found in 86.5% of MSM infected by L2 serovar in Turin [7]. Consistently, a long lasting unrecognized anal syndrome was identified also in the MSM diagnosed with LGV at our center in 2015–2016.

Both in Turin and in our study the majority of LGV cases observed in MSM were diagnosed in HIV-infected individuals on cART (i.e., 95% of cases in Turin and 100% in Rome). The fact that all the cases recently diagnosed have been observed in MSM suggests that anal mucosa could represent an efficient reservoir for CT infection in the MSM community and explain the maintenance of an elevated risk of secondary transmission of infections, particularly among HIV-infected patients.

Notably, the LGV proctitis in our case series was mainly observed in individuals who referred specific receptive anal practices (fisting, sharing of sex toys). As also previously described by others, these sexual practices may play a major role in the spread of STIs among MSM [14, 15]. In fact, they may cause a higher risk of parenteral exposure, thus enhancing the circulation of CT L2b serovar. Sexual behaviour at greater risk is generally reported by “LGV-repeater” patients, i.e., those with LGV re-infections, commonly reported (5.2% of prevalence among all LGV diagnoses) in “endemic” countries, such as UK, and among HIV-positive MSM [16].

In USA and Europe, Chlamydia serology is considered useful to support the diagnosis of LGV in the appropriate clinical context [5]. Nevertheless, to date, there is not unanimous agreement regarding the laboratory methods for LGV diagnosis. Although NAATs are sensitive and specific, there is still a need to identify univocal microbiological approaches. Additionally, in Italy, these methods are not easily available for all STI clinics and some laboratories use home-made CT genotyping tests. We consider the use of these diagnostic tests as mandatory tools for a correct diagnosis, particularly in doubtful and clinically non-specific cases (LGV proctitis or inflammatory bowel disease). In our experience, the increase in the rate of high-risk patients complaining for anal symptoms has led to the use of new methodologies for the molecular diagnosis of CT serovars involved in LGV etiology.

The characteristics of the reported cases of LGV diagnosed in our center seem to suggest a recent increase of circulation of CT L2b serovar infection among MSM. This increase seems to be associated with the spreading of high risk sexual behaviours, particularly among patients living with HIV. Urgent interventions to enhance awareness of the health specialists about LGV are needed. Prevention programmes that involve multidisciplinary teams can be also useful to reduce the number of late diagnoses and to limit LGV transmission.

This study has a few limitations. Firstly, the case series was collected from a single STI center, therefore the number of the cases observed is limited. Secondly, the possibility of sexual contacts among the cases was not investigated. Thirdly, CT infection was not routinely searched for among high-risk individuals attending our STD center. Finally, we cannot exclude the influence of the “clinicians’ attitude” on the trend of LGV cases, i.e., recent awareness of the clinicians about LGV might have led to an increase of the diagnoses. Nonetheless, we believe that our findings still highlight a significant phenomenon, considering the rarity of LGV in developed countries. Consistently with the European guidelines for CT infection control, we sustain the need to investigate for CT anal infection all MSM attending an STI clinic, particularly those who refer any anal discomfort. Moreover, all MSM, particularly those who live with HIV infection, should be continuatively counselled about the risk of some receptive anal practices associated with LGV.

## Abbreviations

BID: Bis in die
cART: Combined anti-retroviral therapy
CT: Chlamydia trachomatis
HIV: Human immunodeficiency virus
LGV: Lymphogranuloma Venereum
MSM: Men who have sex with men
NAATs: Nucleic acid amplification tecniques
PCR: Polymerase chain reaction
STD: Sexually transmitted disease
STI: Sexually transmitted infection
